# Dual Anticancer and Antibacterial Properties of Silica-Based Theranostic Nanomaterials Functionalized with Coumarin343, Folic Acid and a Cytotoxic Organotin(IV) Metallodrug

**DOI:** 10.3390/pharmaceutics15020560

**Published:** 2023-02-07

**Authors:** Maider Ugalde-Arbizu, John Jairo Aguilera-Correa, Victoria García-Almodóvar, Karina Ovejero-Paredes, Diana Díaz-García, Jaime Esteban, Paulina L. Páez, Sanjiv Prashar, Eider San Sebastian, Marco Filice, Santiago Gómez-Ruiz

**Affiliations:** 1 Departamento de Química Aplicada, Facultad de Química, Euskal Herriko Unibertsitatea (UPV/EHU), Paseo Manuel Lardizabal 3, 20018 Donostia San Sebastián, Spain; 2 Clinical Microbiology Department, IIS-Fundación Jiménez Diaz, UAM, Avenida Reyes 15 Católicos 2, 28037 Madrid, Spain; 3 COMET-NANO Group, Departamento de Biología y Geología, Física y Química Inorgánica, ESCET, Universidad Rey Juan Carlos, C/Tulipán s/n, 28933 Móstoles, Spain; 4 CIBERINFEC-CIBER de Enfermedades Infecciosas, 28029 Madrid, Spain; 5 Nanobiotechnology for Life Sciences Group, Department of Chemistry in Pharmaceutical Sciences, Faculty of Pharmacy, Universidad Complutense de Madrid (UCM), Plaza Ramón y Cajal s/n, 28040 Madrid, Spain; 6 Microscopy and Dynamic Imaging Unit, Fundación Centro Nacional de Investigaciones Cardiovasculares Carlos III (CNIC), Calle Melchor Fernandez Almagro 3, 28029 Madrid, Spain; 7 Unidad de Investigación y Desarrollo en Tecnología Farmacéutica (UNITEFA), Consejo Nacional de Investigaciones Científicas y Técnicas (CONICET), Departamento de Ciencias Farmacéuticas, Facultad de Ciencias Químicas, Universidad Nacional de Córdoba, Córdoba X5000HUA, Argentina

**Keywords:** cancer, bacteria, biofilm, oral, topical, luminescence, theranosis, *S. aureus*, biofilm, wound healing

## Abstract

Five different silica nanoparticles functionalized with vitamin B12, a derivative of coumarin found in green plants and a minimum content of an organotin(IV) fragment (**1-MSN-Sn**, **2-MSN-Sn**, **2-SBA-Sn**, **2-FSPm-Sn** and **2-FSPs-Sn**), were identified as excellent anticancer agents against triple negative breast cancer, one of the most diagnosed and aggressive cancerous tumors, with very poor prognosis. Notably, compound **2-MSN-Sn** shows selectivity for cancer cells and excellent luminescent properties detectable by imaging techniques once internalized. The same compound is also able to interact with and nearly eradicate biofilms of *Staphylococcus aureus*, the most common bacteria isolated from chronic wounds and burns, whose treatment is a clinical challenge. **2-MSN-Sn** is efficiently internalized by bacteria in a biofilm state and destroys the latter through reactive oxygen species (ROS) generation. Its internalization by bacteria was also efficiently monitored by fluorescence imaging. Since silica nanoparticles are particularly suitable for oral or topical administration, and considering both its anticancer and antibacterial activity, **2-MSN-Sn** represents a new dual-condition theranostic agent, based primarily on natural products or their derivatives and with only a minimum amount of a novel metallodrug.

## 1. Introduction

A global health problem is the worldwide increase in the incidence of cancer, which is the first cause of death in Europe, the US and China. Furthermore, breast cancer is the most diagnosed and, in particular, invasive ductal carcinoma, which accounts for 80% of all breast cancer cases [1]. Triple negative breast cancer (TNBC) is the most aggressive and has the worst prognosis because it does not respond to hormonal therapy or drugs that target the human epidermal growth factor receptor 2 (HER2) protein and has an overall 5-year survival rate of ca. 12% [2,3].

The increasing emergence of multi-resistant bacterial pathogens in the early 21st century has generated a global health problem. The World Health Organization (WHO) has already underlined the urgency of designing new antimicrobial treatments to affront the problem of conventional antibiotics becoming ever less effective [4,5]. Antibiotic resistance is closely linked to the development of bacterial biofilms [6,7], which constitutes a challenge in the development of efficient treatments to eradicate bacterial infections, particularly in wounds and burns [6,8]. Currently, the incidence of non-healing wounds is like that of heart failure, with antibiotic resistance of biofilms formed by the bacterial species *Staphylococcus aureus* being not only the most common but also the most dangerous one in chronic wounds or prolonged wound healing processes [9,10,11].

It is important to emphasize that there is an intersection between cancer and infection, which is revealed by the number of cancer deaths that are originally derived from chronic infections. Approximately 2.2 million new cancer diagnoses are caused by infectious agents, such as bacteria and viruses [12]. This intersection between cancer and bacterial infections is aggravated by the fact that drug development is long and complex, as the commercialization process can take up to 10–15 years [13]. In this context, the development of dual therapeutic agents with both antimicrobial and anticancer activity is an interesting approach from the point of view of cost/risk in their development and commercialization.

Metal compounds (such as copper and silver) have historically been used against bacterial infections [14,15,16], and since the Food and Drug Administration’s (FDA) approval of cisplatin (1978), the use of metal-based drugs in cancer chemotherapy has also increased considerably [17,18]. Nevertheless, the serious drawback of drug resistance in tumors following treatment with platinum-based compounds has been observed [19] and, therefore, the search for alternative metalloids is currently of strategic importance to circumnavigate cisplatin-related resistance. Among the possible alternatives, organotin(IV) derivatives have emerged as promising candidates due to their potent cytotoxicity against cancer cells and their ability to overcome resistance [20,21,22,23,24]. In addition, organotin(IV) derivatives have been shown to have antibacterial activity [25,26,27], so their use opens the door to the development of dual antibacterial and anticancer drugs.

It is important to note that specificity of such tin-derivatives towards non-healthy cells, can be potentiated via their coadministration with a targeting molecule, such as folic acid (FA), also known as vitamin B12, as folate receptors are overexpressed in many cancer tissues (such as TNBC), as well as on the surface of bacterial cells within biofilms and, therefore, drug uptake should be enhanced upon FA recognition by malignant/bacterial cells [28,29,30,31,32,33,34] FA is a natural molecule found in vegetables and some animal feed, although in spite of its essentiality for nucleotide synthesis, it is not produced de novo by eukaryotic cells and, therefore, needs to be included in the diet [35].

Moreover, not only the specificity of organotin(IV) compounds need to be improved for a potential dual application against cancer and bacterial infections, but also an enhancement of the therapeutic activity, bioavailability and even solubility of the cytotoxic agent and this can be achieved by the use of nanoparticles as drug delivery platforms [36,37]. In this context, silica nanoparticles have emerged as the most widely used and adequate type of carrier, recognized as safe by the FDA for use as oral delivery ingredients [38]. In this context, silica nanoparticles are interesting because of their great abundance, chemical versatility, and high loading capacity of drugs/chemicals/therapeutic molecules, associated with ease of processing [39]. Silica nanoparticles include, among others, mesoporous silica nanoparticles (MSN) and fibrous silica nanoparticles (FSP). These two types of nanoparticles have already been studied as nanocarriers, in the area of oncology and bacterial infections by our team, as the physicochemical parameters can be fine-tuned to achieve the desired behavior and efficient loading and release of the drug at the desired site. Silica-based systems allow control of the pharmacokinetic profile of the transported therapeutic and targeting agents. They tend to preferentially accumulate at sites of tumor growth or inflammation and have a great potential to internalize into cells/bacteria (even in biofilm state) faster than small molecule drugs [40,41,42,43,44,45,46]. Furthermore, the suitability of silica nanoparticles both in topical and oral treatments has been studied, demonstrating excellent biocompatibility [47,48,49,50,51].

In addition to the need for a potent biological activity and a large therapeutic application, it is advantageous that the material shows theranostic properties [52,53], with the ability both to eradicate and to diagnose a disease or its progression. In this context, biomedical imaging techniques can be exploited for disease diagnosis, especially in cancer related diseases, both pre- and post-intervention [54,55,56]. Imaging techniques, such as fluorescent imaging, requires the administered therapy to contain a fluorophore able to emit visible light once irradiated with the appropriate lamp. In the case of functionalized silica nanoparticles as drug delivery platforms, the particles can easily be further decorated with luminescent diagnostic molecules. Among the large number of fluorophores, derivatives of the naturally occurring coumarins are particularly suitable. Coumarins consist of natural benzopyrenes identified primarily in green plants [57] and, not only do they possess appropriate luminescent properties for in vivo imaging, but they have been shown to be cytotoxic to cancers, such as TNBC expressing the monocarboxylate transporter genes 1 and 4 (MCT1 and MCT4), and to have the ability to suppress bacterial quorum sensing, thus preventing the formation of biofilms [58,59,60,61]. Therefore, a theranostic drug delivery platform where coumarin derivatives are used as fluorophores, may also benefit from an increased therapeutic activity due to this secondary cytotoxic role.

In this study, a series of five new multifunctionalized silica nanoparticles were synthesized, characterized, and tested as potential dual anticancer and antibacterial agents. Four types of silica-based nanocarriers (MSN, SBA-15 and 2 types of FSP) were functionalized with: (i) an organotin(IV) complex as the main cytotoxic agent, (ii) FA as a natural targeting molecule and (iii) a derivate of natural coumarin (coumarin343) as a diagnostic fragment and a secondary cytotoxic agent. In vitro cytotoxicity against the breast cancer cell line MDA-MB-231 as well as antibacterial activity against *Staphylococcus aureus* planktonic cultures and biofilms was determined. In addition, a complete study of the NP-biofilm interaction, attachment, effect on biofilm development and ROS generation was performed, as well as cellular uptake/compound internalization confirmed by imaging techniques both in cancer cells and biofilms.

## 2. Materials and Methods

### 2.1. General Remarks on Characterization of the Materials

For the characterization of the nanomaterials, transmission electronic microscopy (TEM) measurements were carried out on a TECNAI G2 20 TWIN operated at 200 kV and equipped with LaB6 filament and high angle annular dark-field-scanning transmission electron microscopy (HAADF-STEM). TEM samples were prepared using a dispersion in ethanol applying ultrasounds for 15 min and adding a drop of the suspension to a TEM copper grid (300 Mesh) covered by a holey carbon film and drying the grid at room temperature. The micrographs were analyzed with ImageJ^©^. Textural properties of samples were obtained by N_2_ physisorption in Micromeritics ASAP 2020. Samples were degassed at 180 °C applying vacuum of 10 μm Hg during 8 h. The materials were then measured at −196 °C. Brunauer Emmett Teller (BET) and Barrett–Joyner–Halenda (BJH) studies of the desorption branch were used to determine the surface area and the pore size distribution. Inductively coupled plasma mass spectrometry (ICP-MS) measurements were recorded using an Agilent 7700 spectrometer, while a thermogravimetric analysis (TG) was carried out using a TG-Q500 TA Instrument thermal analyzer from 20 to 750 °C with a heating rate of 10 °C min^−1^ and using a nitrogen atmosphere. The powder X-ray diffraction (XRD) patterns were collected on a Phillips X’PERT powder diffractometer with CuKα radiation (λ = 1.5418 Å) in the following ranges: 0.8 < 2θ < 10°, and with a step size of 0.026° with an acquisition time of 2.5 s per step at 25 °C. Fourier Transformed-infrared (IR) spectra (400–4000 cm^−1^) were recorded on a Nicolet FT-IR 6700 spectrometer using KBr pellets. DR-UV measurements were carried out using a UV/Vis Shimadzu spectrophotometer. The spectra were obtained at room temperature using BaSO_4_ as the reference material. ^13^C CP MAS NMR Solid-State nuclear magnetic resonance (NMR) measurements were carried out in a high-resolution mode, at 298 K on a Bruker Avance 400 WB spectrometer at 9.4 T, using 400.17 (^1^H) and 100.66 MHz (^13^C) resonance frequencies. The ^13^C NMR experiments were recorded using a cross-polarization (CP) technique, high power decoupling, and magic angle spinning (MAS) with rates of 10 kHz, using a Bruker double-bearing probe head and 4 mm zirconia rotors driven by dry air. The Hartmann–Hahn conditions for ^13^C NMR were matched using adamantane. The recycle delay was 5 s and the contact time was 2 ms. Chemical shifts were determined by using an external standard based on glycine (Gly) (dCO of Gly= 176.5 ppm). Photoluminescence (PL) measurements were recorded at room temperature with a Varian Cary-Eclipse fluorescence spectrofluorometer with a Xe discharge lamp (peak power equivalent to 75 kW), Czerny–Turner monochromators, and an R-928 photomultiplier tube. The measurements were carried out at a photomultiplier detector voltage of 600 V, and with both the excitation and emission slits set at 5 nm.

### 2.2. Synthesis of the Starting Silica Materials (MSN and SBA-15)

MSN nanoparticles were prepared using the method published by Zhao and coworkers [62]. In summary: 2.74 mmol of CTAB (cetyltrimethylammonium bromide) was dissolved in 480 mL of water; subsequently, a solution of NaOH (2.0 M, 3.5 mL) was added dropwise at room temperature and the mixture heated to 80 °C. Then, 5 mL (22.4 mmol) of the silica precursor TEOS (tetraethyl orthosilicate) was added dropwise and left under vigorous stirring for 2 h. The white precipitate was filtered and washed with abundant water and methanol and dried for 24 h at 80 °C in an oven. Finally, the sample was calcined at 550 °C for 24 h.

The synthesis of SBA-15 was carried out with a modification of the method described by Zhao et al. [63]. Briefly, 24 g (4.14 mmol) of the surfactant Pluronic 123 was dissolved in 180 mL of Milli-Q water and 730 mL of a 2 M solution of hydrochloric acid. Under vigorous stirring, 54.66 mL (24.5 mmol) of the silica precursor TEOS was added dropwise. The mixture was kept at 35 °C for 20 h. After that time, the stirring was stopped, and the temperature was increased to 80 °C for an additional 24 h. The resulting suspension was filtered, and the isolated solid washed with abundant water and methanol and calcinated under the same conditions as MSN.

FSPm and FSPs were purchased from Strem Chemicals Inc. (Newburyport, MA, USA) and used directly, without prior purification.

### 2.3. Functionalization of Silica Materials with Amino Ligand Synthesis of SiO_2_-AP

**Scheme 1:**
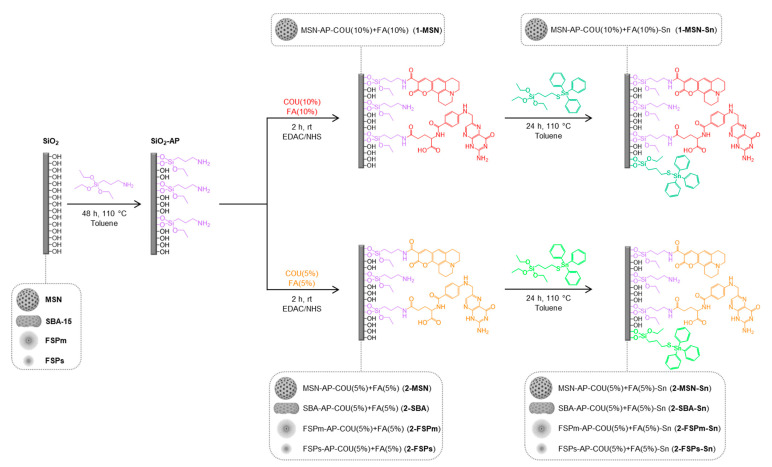
Synthetic routes in the preparation of the functionalized materials.

For the incorporation of amino groups into the silica materials, the ligand (3-aminopropyl) triethoxysilane (AP) was used (Scheme 1). The silica particles were dispersed in dry toluene and a proportion of 10% AP/SiO_2_ *w*/*w* added as the optimal quantity of functionalization [64]. The solution was kept under stirring at 110 °C for 48 h. Finally, the suspension was centrifuged (6000 rpm, 10 min), washed with toluene and diethylether, and dried in a stove. The materials obtained were named MSN-AP, SBA-AP, FSPm-AP, and FSPs-AP.

### 2.4. Incorporation of the Targeting and Imaging Agents

Coumarin343 (COU) was used as imaging fluorophore and folic acid (FA) as the targeting agent. **MSP-AP** was functionalized with either 5% or 10% *w*/*w* SiO_2_/COU and FA (Scheme 1). For FA and COU incorporation, an EDAC coupling process in MES buffer was performed. For the coupling reaction a solution of COU and FA in DMSO was added to the MES buffer with EDAC and NHS in a molar proportion of 1:2.5, for the 5% and 10% load, respectively. The mixture was stirred for 15 min at room temperature and subsequently **MSN-AP** was added. The resulting suspension was maintained under stirring for 2 additional hours at room temperature. Finally, the solid was isolated by centrifugation (6000 rpm, 10 min) and the isolated solid was washed with dimethylsulfoxide (DMSO) and water, obtaining the materials MSN-AP-COU(10%) + FA(10%) (**1-MSN**), MSN-AP-COU(5%) + FA(5%) (**2-MSN**), SBA-AP-COU + FA (**2-SBA**), FSPm-AP-COU(5%) + FA(5%) (**2-FSPm**) and FPSs-AP-COU(5%) + FA(5%) (**2-FSPs**).

### 2.5. Synthesis and Incorporation of the Cytotoxic Agent

The preparation of the organotin(IV) compound Ph_3_Sn{SCH_2_CH_2_CH_2_Si(OEt)_3_} (MP-Sn) was carried out following the procedure described previously by our group [64]. In a Schlenk tube, SnPh_3_Cl was dissolved in dry toluene and triethylamine and 3-mercaptopropyltriethoxysilane (MP) were added in a molar proportion of 1:2:1, respectively. The mixture was stirred at 110 °C for 24 h. The reaction was then stopped, and MP-Sn isolated by filtration.

A solution of MP-Sn in toluene was added to a dispersion of the silica material in dry toluene (functionalization ratio 10% Sn: SiO_2_ w/w). The final mixture was maintained at 110 °C for 24 h (Scheme 1). The final materials were centrifuged and the isolated solid washed with toluene and ethanol and dried in a stove. The codes for these materials are MSN-AP-COU(10%) + FA(10%)-Sn (**1-MSN-Sn**) for the material with 10% of COU and 10% of FA of functionalization and MSN-AP-COU(5%) + FA(5%)-Sn (**2-MSN-Sn**), SBA-AP-COU(5%) + FA(5%)-Sn (**2-SBA-Sn**), FSPm-AP-COU(5%) + FA(5%)-Sn (**2-FSPm-Sn**) and FPSs-AP-COU(5%) + FA(5%)-Sn (**2-FSPm-Sn**) for the materials with 5% of COU and 5% of FA.

### 2.6. In Vitro Studies in Cancer Cells

#### 2.6.1. Cell Culture

Triple-negative human breast adenocarcinoma cell line, MDA-MB-231 acquired from Innoprot (Derio, Bizkaia, Spain and provided through Dismadel SL, Madrid, Spain), was grown in Dulbecco’s Modified Eagle Medium (DMEM)-F12 supplemented with 10% heat-inactivated Fetal Bovine Serum (FBS), 1% non-essential amino acids, 1% sodium pyruvate 100 mM and 1% penicillin/streptomycin. Human embryonic kidney cells, HEK-293T acquired from the American Type Culture Collection [ATCC] (Manassas, VA, USA, REF: CRL-3216), were grown in DMEM with GlutaMAX^TM^-L supplemented with 10% FBS, 1% sodium pyruvate 100 mM and 1% penicillin/streptomycin. The cells were maintained at 37 °C in a humidified atmosphere with 5% CO_2_.

#### 2.6.2. Cell Viability Assays

For the cytotoxicity assays, the cells were cultivated on 96-well plates, at a concentration of 7.5 × 10^3^ cells/mL in 100 μL of media in each well for two days. After that time, the cell culture media was removed, and the wells were treated with dispersions of each silica-functionalized material from 0.1 µM to 100 µM, for 24 h. After incubation, the dispersions were discarded and replaced by 100 μL of medium, without phenol red and without serum, and 10 μL of a 12 mM MTT (dimethylthiazolyl-diphenyl-tetrazolium bromide) solution was added to each well and mixed. After 3 h of incubation, all the supernatants less 25 μL were removed and 100 μL DMSO was added to each well to dissolve the formazan, leaving it 15 min to react. A negative control of cells incubated with media and without any material, and positive control incubating cells with 1:1 vol of DMSO/media solution were also tested. Cell viability was measured with the absorbance at 570 nm using a SPECTROstar Nano plate reader (BMG Labtech Inc., Cary, NC, USA). IC_50_ values for materials containing tin have been referred to in terms of the concentration of metal in each material. To assess whether the toxicity is due to the metallodrug or the silica nanoparticles itself, materials of the same concentration of silica were compared with and without the cytotoxic organotin(IV) compound.

#### 2.6.3. Cellular Uptake

A total of 3 × 10^5^ MDA-MB-231 cells were seeded in a 6-well culture plate and incubated overnight with the nanomaterials (**2-MSN-Sn**, **2-SBA-Sn**, **2-FSPm-Sn** and **2-FSPs-Sn**) at a final tin concentration of 0.1 µM. Wells were washed with phosphate buffered saline (PBS) 1× and cell culture medium without phenol red was added. Then, images were taken using a confocal microscope Zeiss LSM 780 (20× water objective) at 37 °C and under 5% CO_2_. Fluorescence images of coumarin (ex/em 443/480 nm) were taken with a 405 nm laser line, and processed with ImageJ Fiji and Imaris software.

### 2.7. In Vitro Studies in Bacteria

An American Culture Collection (ATCC29213) strain of *Staphylococcus aureus* was studied. The strain was stored frozen at −80 °C until experiments were performed.

#### 2.7.1. Minimum Inhibitory Concentration and Minimum Bactericidal Concentration

The minimum inhibitory concentration (MIC) of each final material was determined according to the standardized Clinical and Laboratory Standards Institute Microdilution method [65]. The inoculum was prepared from overnight cultures with a cell concentration of 1.6 × 10^8^ colony forming unit per mL (CFU/mL) corresponding to 0.5 on the McFarland scale. It was then diluted 1:100 in Mueller Hinton Broth (MHB) medium. In a 96-well microplate, serial dilutions were performed from 2000 to 1.9531 µg/mL and then the inoculum was added at the concentration of 1.6 × 10^6^ CFU/mL. Finally, it was incubated for 18 h at 37 °C and 5% CO_2_ and developed using MTT (3-(4,5-dimethyl-2-thiazolyl)-2,5-diphenyltetrazole bromide)) as the developing agent. MIC was defined as the lowest concentration where no bacterial growth was observed after at least 18 h incubation.

The minimum bactericidal concentration (MBC) was determined using the Flash Microdilution method described above with some modifications [66]. In summary, 20 μL of each well after 24 h incubation was mixed with 180 μL of tryptic soy broth (TSB) in a new 96-well plate and statically incubated at 37 °C and 5% CO_2_ for 24 h. The MBC was defined as the lowest concentration where no turbidity occurred, measuring the absorbance at 600 nm using a Tecan Infinite M200 Plate Reader.

#### 2.7.2. Attachment of Materials to Bacteria

Quantification of attachment of materials to bacteria by fluorometry was determined using previously described methodology [67]. To analyze the internalization of the synthesized **2-MSN** and **2-MSN-Sn** materials, a bacterial suspension of *S. aureus* ATCC29213 was prepared in TSB. The bacterial cells were incubated with 20 µg/mL of each compound for 1 h. Subsequently, the samples were centrifuged at 2000× *g* at room temperature and the supernatant was separated. Fluorescence was determined on both samples in a Synergy HT microplate reader (BioTek, Winooski, VT, USA). The sediment was then resuspended in phosphate buffer (PBS) pH 7.2 and sonicated for 30 min to induce bacterial lysis. The samples were then centrifuged, and the supernatant separated for fluorescence determination (excitation 490 nm, emission 520 nm).

#### 2.7.3. Study of Oxidative Stress

Oxidative stress was followed by reduction of nitrotetrazolium blue (NBT). This test is based on the reduction of oxidized (colorless) NBT to reduced NBTH, as precipitate (formazan blue). The assays were performed with *S. aureus* ATCC29213. In summary, 0.1 mL of bacterial suspension (OD_600_ = 1) in PBS pH 7 was incubated for 0, 1 and 4 h with 0.1 mL of **2-MSN** or **2-MSN-Sn** at MIC/10 (12.5 µg/mL), MIC (125 µg/mL) and MICx10 (1250 µg/mL) concentrations for *S. aureus*; the material was replaced by PBS in control samples. Then, the cells were centrifuged for 10 min at 1500 rpm to separate the cells from the supernatant (extracellular ROS). Then, 0.5 mL of NBT (Sigma) 1 mg/mL was added and the samples were incubated for 30 min at 37 °C. Subsequently, 0.1 mL of 0.1 M HCl (Cicarelli) was added to stop the reaction. Cell pellets were treated with 0.4 mL DMSO (Cicarelli) to remove the reduced NBT (intracellular ROS) and then 0.8 mL PBS was added. Formazan blue was quantified spectrophotometrically at 575 nm. The experiment was performed in triplicate.

#### 2.7.4. *S. aureus* Biofilm–Nanoparticles Interaction

To study the interaction between nanoparticles and biofilms of *S. aureus* ATCC29213 strain, a previously described methodology was used with some modifications [68]. In total, 200 µL of 1.6 × 10^6^ CFU/mL in TSB + 1% glucose was placed in a 96-well flat-bottom plate and statically incubated at 37 °C and 5% CO_2_ for at least 18 h. After incubation, the supernatant was removed, and each well was washed twice using 200 µL of saline 0.9% NaCl. Then, 200 µL of saline without NP as control and with 250 µg/mL of each type of nanoparticle to be studied (**MSN**, **2-MSN** and **2-MSN-Sn**) were placed in the wells. They were then incubated at 100 rpm, 37 °C and 5% CO_2_ for 30 min. Finally, each well was rinsed again with 200 μL of saline and stained with 2% crystal violet, according to a previously reported methodology [69]. The absorbance of the system was measured at 570 nm using a Tecan Infinite M200 Plate Reader. The experiment was performed in triplicate (*n* = 24 per condition).

#### 2.7.5. Inhibition of Adherence Stage during the Biofilm Formation

A P96 plate was used to study the inhibition of the initial biofilm attachment stage of *S. aureus* ATCC29213. *S. aureus* bacterial suspensions (1.00 ± 0.02 McFarland turbidity scale, approximately 1.6 × 10^8^ CFU/mL) were prepared for each type of nanoparticle to be studied (**MSN**, **2-MSN** and **2-MSN-Sn**) with a final concentration of 250 µg/mL. The bacterial suspension without nanoparticles was used as control. A total of 200 µL of these bacterial suspensions was deposited on the P96 plate and statically incubated at 37 °C and 5% CO_2_ for 1.5 h. After incubation, each well was washed twice with 200 µL of saline, filled with 200 µL of TSB + 1% glucose and incubated at 37 °C and 5% CO_2_ for 24 h. Finally, they were developed using MTT. For this, 20 µL of MTT was added to each well and the plate was shaken at 90 rpm in an incubator at 37 °C and 5% CO_2_ for 1 h. After incubation, absorbance was measured at 570 nm using a Tecan Infinite M200 Plate Reader. The experiment was performed in triplicate (*n* = 24 per condition).

#### 2.7.6. Effect on Biofilm Development

To study whether the materials inhibit biofilm development, a 96-well plate was used for *S. aureus* ATCC29213. The materials used were **MSN**, **2-MSN** and **2-MSN-Sn**. The concentration used for each material was 2 × MIC of the tin-containing material. For this purpose, 200 µL of 1 × 10^6^ CFU/mL in TSB + 1% glucose of each strain with a 2 × MIC concentration of the material was deposited in a 96-well flat-bottom plate and incubated at 37 °C and 5% CO_2_ for 24 h. After incubation, each well was rinsed twice with 200 µL of saline. Biofilm quantification was performed according to previously reported methodology [69]. In summary, each well was fixed with 200 µL of MeOH and kept for 20 min in air, the supernatant was then removed and allowed to dry at 60 °C. After fixation, staining was performed. For this purpose, 150 µL of 2% crystal violet was added and 15 min were given to allow the dye to penetrate through the biofilm. Each well was then rinsed twice with 200 µL of distilled water and solubilized with absolute ethanol. Finally, absorbance was measured at 570 nm in a colorimeter using a Tecan Infinite M200 Plate Reader. This experiment was performed in eight wells for each material and strain and in triplicate (*n* = 24).

#### 2.7.7. Optical and Fluorescence Microscopy

For visualization of the inhibition of biofilm development by optical and fluorescence microscopy, 200 µL of 1 × 10^6^ CFU/mL *S. aureus* ATCC29213 in TSB + 1% glucose with a 2 × MIC concentration of the **2-MSN-Sn** material was deposited in a 24-well flat-bottomed plate with a sterile glass cover and incubated at 37 °C for 24 h. An untreated bacterial suspension was used as control. Fluorescence microscopy photos were taken on a Nikon TE-2000U 40X microscope (Tokyo, Japan) (excitation 490 nm; emission 519 nm; mirror 500 nm; emission LP 515 nm). After incubation, each well was rinsed twice with PBS. After rinsing each cover, staining was performed. For this, 500 µL of 1% crystal violet was added and left for 20 min to allow the dye to penetrate through the biofilm and then dried at room temperature. Finally, photographs were taken by optical microscopy with an immersion objective (100×).

#### 2.7.8. Statistical Analysis

Statistical analyses were performed using GraphPad Prism 8 software (version 8.01, GraphPad, San Diego, CA, USA). Data were evaluated using a two-sided nonparametric Mann–Whitney test to compare two groups and nonparametric Kruskal–Wallis test to compare more than two groups. Statistical significance was set at *p*-values ≤ 0.05.

## 3. Results and Discussion

### 3.1. Synthesis and Physicochemical Characterization of Functionalized NPs

All materials were fully characterized by transmission electronic microscopy (TEM), thermogravimetry analysis (TG), inductively coupled plasma mass spectrometry (ICP-MS), Fourier transformed-infrared spectroscopy (FT-IR), diffuse reflectance ultraviolet spectroscopy (DR-UV), X-ray diffraction (XRD), nitrogen adsorption-desorption isotherms, solid-state ^13^C CP MAS NMR spectroscopy and photoluminescence.

#### 3.1.1. Analysis of Size, Morphology, and Textural Properties

**Figure 1:**
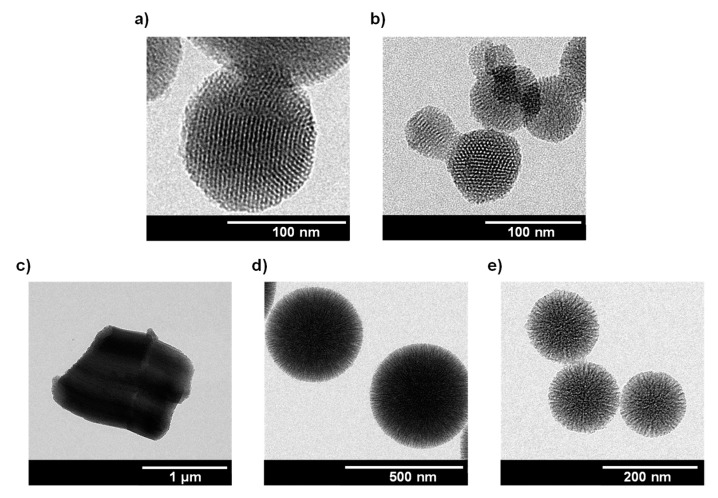
TEM images of (**a**) 1-MSN-Sn, (**b**) 2-MSN-Sn, (**c**) 2-SBA-Sn, (**d**) 2-FSPm-Sn, and (**e**) 2-FSPs-Sn.

Morphology and particle size of each final material was determined from the TEM micrographs (Figure 1). **1-MSN-Sn** and **2-MSN-Sn** consist of spherical structures with hexagonally arranged porous parallel channels. Particle sizes (for particle size distributions see Figure S1 in SI) were found to be 132.7 ± 28.7 nm for **1-MSN-Sn** and 85.8 ± 24.5 nm for **2-MSN-Sn**. In the case of **2-SBA Sn**, hexagonal particles and well-ordered mesoporous channels 1054.0 ± 145.0 nm in length are observed (Figure S1). As for the fibrous silica particles, both **2-FSPm-Sn** and **2-FSPs-Sn** present a fibrous spherical shape with a particle size of 449.7 ± 43.5 nm and 148.6 ± 13.9 nm (Figure S1), respectively.

The characterization of the textural properties, namely, surface area (BET) and pore volume and diameter, were estimated both for the starting and final materials through the analysis of nitrogen adsorption–desorption isotherms (see Figures S2 and S3 in SI). Textural property data derived from such isotherms are collected in Table 1.

In the case of **MSN** and **MSN**-based materials, and according to the IUPAC classification, all isotherms can be classified between type IV and type VI [70]. The isotherms showed a hysteresis loop between P/P_0_ 0.90–1.0, originated mainly by the capillary condensation of nitrogen into the pore of the system.

In addition, a decrease in BET surface area was observed when the unmodified **MSN** material (1042 m^2^·g^−1^) was functionalized with the organotin fragment (**1-MSN-Sn** 691 m^2^·g^−1^ and **2-MSN-Sn** 702 m^2^·g^−1^), which is indicative of the functionalization occurring inside the pore which induces a decrease in pore volume and diameter.

**SBA-15** and **SBA**-based organotin functionalized materials exhibited type IV isotherms according to the IUPAC classification [70]. Both displayed a H2b type hysteresis loop between 0.50 and 0.70 relative pressures, reflecting the typical capillary condensation process of mesoporous materials. **SBA-15** showed a superficial area of 650 m^2^·g^−1^ and a BJH pore diameter of 4.74 nm.

**FSPm** materials isotherms were between type IV and VI and display hysteresis loops. In the case of unmodified **FSPm**, the hysteresis loop is larger than for **FSPm**-based final material. **FSPm** hysteresis is observed between P/P_0_ 0.2 and 0.8 but it down-shifts to ca. 0.4 in the case of **2-FSPm-Sn**. Both hysteresis loops are associated with capillary condensation of nitrogen into the pores. Furthermore, superficial area and pore volume decreased by almost half from the starting material to the organotin functionalized one, from 317 to 132 m^2^·g^−1^ and from 0.49 to 0.23 cm^3^·g^−1^, respectively.

Finally, the isotherms of starting **FSPs** and modified materials display type IV isotherms with two hysteresis loops between P/P_0_ about 0.40–0.60 and 0.90–1.0; again, most probably due to capillary condensation. The adsorption analysis allowed the determination of the BET surface areas of **FSPs** and **2-FSPs-Sn**, which were 315 and 168 m^2^·g^−1^, respectively. Additionally, the pore volume and pore size decreased.

In all the cases, the decrease in BET surface areas of final functionalized materials is indicative of the functionalization occurring inside the pores, which also provokes a decrease in pore volume and diameter.

#### 3.1.2. Quantification of the Degree of Functionalization by Thermogravimetry and Inductively Coupled Plasma Mass Spectroscopy

In order to determine the actual degree of functionalization of the materials (incorporation of linker ligand and cytotoxic, targeting and imaging agents), TG and ICP-MS techniques were employed. The quantification of AP ligand and COU + FA was carried out by TG observed mass loss occurring between 125 and 650 °C (Figure S4). Tin quantification was determined through ICP-MS upon sample digestion in basic medium. The incorporation of the cytotoxic agent MP-Sn was quantified according to the percentage of tin in the final materials.

As observed in Table 2, both AP ligand degree of functionalization and EDAC coupling of coumarin + folic acid was higher for those silicas with larger superficial areas and ordered pore distribution (**MSN** and **SBA-15**). The highest tin content was found in the final material **2-MSN-Sn**, up to four times higher than for other final systems, probably due to an appropriate combination of superficial area and morphology, with the ordered internal distribution. With respect to fibrous silica particles, **2-FSPs-Sn** incorporated 1.5 times more tin than its analogous **2-FSPm-Sn**. Therefore, morphological and size differences of the silica influence the yield of successive functionalization steps.

#### 3.1.3. Characterization by Powder X-ray Diffraction Studies

**Figure 2:**
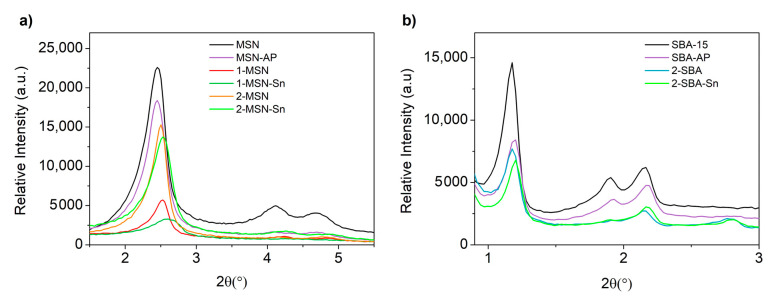
XRD diffractograms of (**a**) **MSN**-based and (**b**) **SBA-15**-based materials.

**MSN** and **SBA-15** starting materials and the corresponding functionalized materials have been characterized by XRD (Figure 2 and Table 3). The unmodified materials **MSN** exhibited three peaks at 2θ of 2.45°, 4.12° and 4.67°, corresponding to (100), (110) and (200) Miller planes, respectively. The relatively high intensity of the first peak (100) is typical in hexagonally ordered mesoporous materials. For the unmodified **SBA-15** material, typical signals associated with hexagonally ordered silica were observed at 2θ of 1.18°, 1.90° and 2.17°, corresponding to the (100), (110) and (200) Miller planes, respectively. Diffraction peaks of the functionalized materials are slightly shifted to higher angles and intensities decreased. This is usually associated with pore size decrease and the partial blocking of the dispersion points of the porous system caused by the incorporation of the organic and/or organometallic fragments inside the pores, respectively.

The successful functionalization of the materials was further confirmed by FT-IR, diffuse reflectance UV-visible spectroscopy and solid-state NMR spectroscopic studies (see details in SI, Figures S5–S7).

### 3.2. In Vitro Studies of Antibacterial and Anticancer Activity

#### 3.2.1. Cell Viability Studies in Cancer Cells

MDA-MB-231 cancer cell line viability after incubation with **1-MSN-Sn**, **2-MSN-Sn**, **2-SBA-Sn**, **2-FSPm-Sn**, **2-FSPs-Sn** and their corresponding starting materials **MSN**, **SBA-15**, **FSPm** and **FSPs** was determined through the half maximal inhibitory concentration (IC_50_) obtained with the MTT cell viability assay (Figure S10, Table 4 and Table S1). In addition, the same experiments with the healthy cell line HEK-293T (Figure S11, Table 4 and Table S1), which have a relatively low folate receptor expression, were performed [71].

As derived from data collected in Table 4, all tin materials showed significant cytotoxicity against MDA-MB-231 cells, with IC_50_ values in the very low or low micromolar range (with respect to the quantity of tin). The cytotoxicity of **2-MSN-Sn** (IC_50_ 0.69 ± 0.31 µM) was found to be more than 11 times higher than that of **1-MSN-Sn** (IC_50_ 7.73 ± 0.24 µM). This probably derives from the higher functionalization of the latter, which makes the cytotoxic fragment less accessible compared to the material with a lower amount of COU + FA.

In addition, **2-MSN-Sn** is significantly more active than **1-MSN-Sn**, and that activity against MDA-MB-231 cancer cells is higher than over healthy HEK-293T cells. A theoretical functionalization degree of a 5% of COU + FA was determined to be the best composition for **MSN** based materials, and hence this was also the value used for the functionalization of **SBA-15**, **FSPm** and **FSPs**-based materials.

**2-SBA-Sn, 2-FSPm-Sn** and **2-FSPs-Sn** functionalized with COU + FA showed biological activities like **2-MSN-Sn**; the most active material was **2-FSPm-Sn**, with an IC_50_ value of 0.44 ± 0.03 µM. However, **2-FSPm-Sn** is even more toxic (IC_50_ value of 0.18 ± 1.06 µM) against kidney cells, a fundamental secretion pathway for nanoparticle elimination. The nanomaterial **2-SBA-Sn** showed a low IC_50_ value (0.81 ± 0.05 µM) against cancer cells and significantly less toxicity (IC_50_ value of 5.31 ± 1.94 µM) towards HEK-293T cells. Remarkably, these materials exhibit higher activities than similar materials previously tested in our group [64,72], and more importantly, these improvements were achieved with a lower organotin(IV) content. The high activity of the systems was probably achieved through the incorporation of coumarin343 into the materials, as this compound may also play a cytotoxic role against MDA-MB-231, a cell line that overexpress the MCT1 and MCT4 genes [58,59,60].

Taken together, the comparison of cytotoxic results between cancer and non-cancer cells, and between the different materials clearly indicate **2-MSN-Sn** as the best drug candidate, with IC_50_ = 0.69 ± 0.31 µM for MDA-MB-231 cells (4.51 ± 1.78 µg/mL based on silica concentration) and IC_50_ =1.63 ± 0.52 µM for HEK-293T cells, equaling the data reported for the standard-of-care 5-fluorouracil (IC_50_ of 3.88 µg/mL) [73]. Both the low tin content and comparatively better results makes **2-MSN-Sn** a very promising agent to be further explored in the treatment of TNBC.

#### 3.2.2. Minimum Inhibitory Concentration (MIC) and Minimal Bactericidal Concentration (MBC)

Once the anticancer activity of the systems was demonstrated, the antibacterial effects of the final materials were evaluated by studying the MIC and MBC values upon incubation of planktonic *S. aureus* ATCC29213 with different concentrations of the final materials. The results collected in [74].

Table 5 show that **2-MSN-Sn** is the only material with antibacterial activity against *S. aureus*, with a MIC value of 125 µg/mL or 2.5 µg/mL considering the material or tin content, respectively. These values are like those reported for clinically used antibiotics, such as ciprofloxacin, erythromycin, gentamicin, tetracycline, doxycycline and chloramphenicol (with MIC values between 0.5 and 128 μg/mL against *S. aureus*, for the comparison with the specific strain see Table S2 of Supplementary Materials) [75]. In addition, and even though the MBC value for **2-MSN-Sn** was high (2000 µg/mL for the material and 40.0 µg/mL for the tin centers), it was superior to that reported for similar MSN materials previously published by our group [74].

All these results point to the **2**-series as promising anticancer agents, but only **2-MSN-Sn** as an antibacterial agent; therefore, its cellular uptake both in bacteria and cancer cells was followed by imaging techniques, to determine its potential use as a dual-condition theranostic agent.

#### 3.2.3. Internalization of Materials in Bacteria

**Figure 3:**
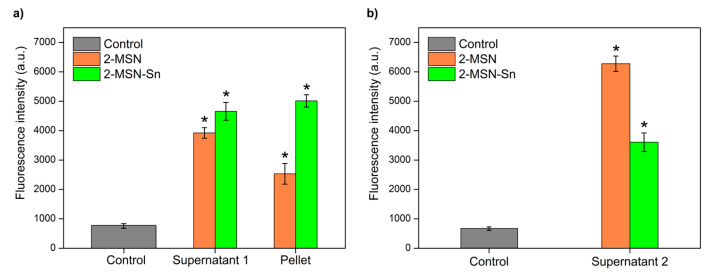
Internalization experiment. Fluorescence intensity of the cell and (**a**) supernatant and pellet before the lysis process and (**b**) pellet supernatant after the lysis process. The study was performed in triplicate and the data are expressed as mean ± SD. * *p*-value < 0.05 respect to control.

Coumarin content inside and outside the cells before (control) and after bacterial incubation with **2-MSN-Sn** was estimated by comparing fluorescent intensities. Upon incubation with **2-MSN-Sn**, cells were pelleted (Pellet) and the supernatants collected before (supernatant 1) and after (supernatant 2) bacterial lysis. Figure 3 shows the fluorescence intensity which is dependent on the amount of compound internalized in the bacteria.

Thus, by observing the values between the intensities of the same compound (Figure 3a), it follows that compounds **2-MSN** and **2-MSN-Sn** interact differently with bacteria. Before the lysis process, the **2-MSN** material interacted less with the pellet, because, after the centrifugation process, ca. 39% of the material remained in the bacterial pellet and the remaining in the supernatant, while the **2-MSN-Sn** compound remained at around 52% in the pellet. In contrast, after inducing lysis of the bacteria (Figure 3b), it was observed that the **2-MSN** material showed higher internalization than **2-MSN-Sn**, as the intensity of the pellet supernatant was higher. **2-MSN-Sn** might be more associated with the bacterial wall than the material without tin. This difference could be related to the fact that the **2-MSN-Sn** material, unlike **2-MSN**, contains the organotin(IV) complex, thus hindering the full accessibility of folic acid reducing the attachment of the compounds on bacteria. These results support a possible interaction between folic acid and the *S. aureus* surface as other authors previously reported [76]. Furthermore, the nanosystem here described might be used as a nanocarrier against *S. aureus*-infected tissues as folate functionalized lipid nanoparticles have shown to be a specifically targeted therapy of methicillin-resistant *S. aureus*-infected tissues [77]. Nevertheless, another possible explanation for this observation is as **2-MSN-Sn** material contains tin, it can have more interaction with the cell membrane [26], which prevents effective internalization.

#### 3.2.4. Study of Oxidative Stress

Oxidative stress is caused by a high concentration of reactive oxygen species (ROS) and is often behind the toxicity of many antibiotics [78]. To determine whether the mode of action of **2-MSN-Sn** in *S. aureus* proceeds via production of ROS, the increase both at intra (ROS_intra_) and extra-cellular (ROS_extra_) levels was determined and quantified at different time (0 h, 1 h and 4 h) and concentration intervals (MIC/10, MIC, and MIC × 10).

**Figure 4:**
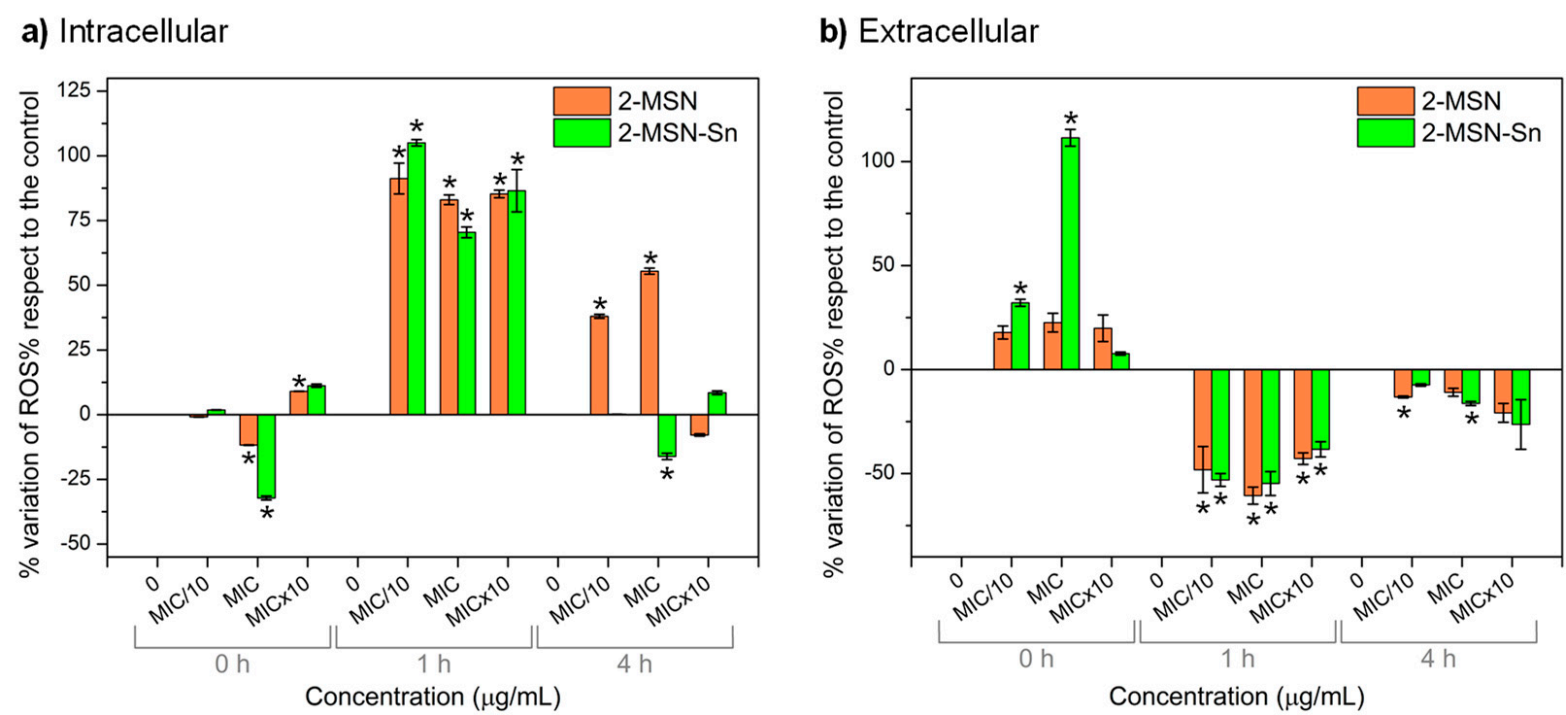
Percentage of variation of (**a**) intracellular and (**b**) extracellular ROS induced by **2-MSN** and **2-MSN-Sn** materials in *S. aureus* at 0, 1 and 4 h of incubation and at three material concentrations. The study was performed in triplicate and the data are expressed as mean ± SD. * *p*-value < 0.05 respect to control.

Immediately after (0 h) incubation with either **2-MSN-Sn** or **2-MSN**, ROS were released to extracellular environment as observed in Figure 4a,b, where % of intracellular and extracellular, respectively, ROS variation with respect to control is represented. In contrast, intracellular ROS dramatically increased after 1 h of incubation both with **2-MSN-Sn** or **2-MSN**. In this respect, the former, even at a sub-MIC concentration (MIC/10 = 12.5 µg/mL), triggered a 105 ± 1% ROS increase as compared to the control, whereas the latter led to a significant increase ca. 85–90% at all the studied concentrations (MIC/10, MIC and MIC × 10). After 4 h of incubation, both intra and extracellular ROS levels dramatically decreased even at a sub-MIC concentrations of **2-MSN-Sn**, but only at a supra-MIC concentration of **2-MSN,** due to the ability of **2-MSN-Sn**, but not so much of **2-MSN**, to lower cell viability [79,80]. In this context, it is very important to consider the potential action of antioxidant systems which give some insights that may support this behavior [81,82].

#### 3.2.5. *S. aureus* Biofilm–Nanoparticles Interaction

**Figure 5:**
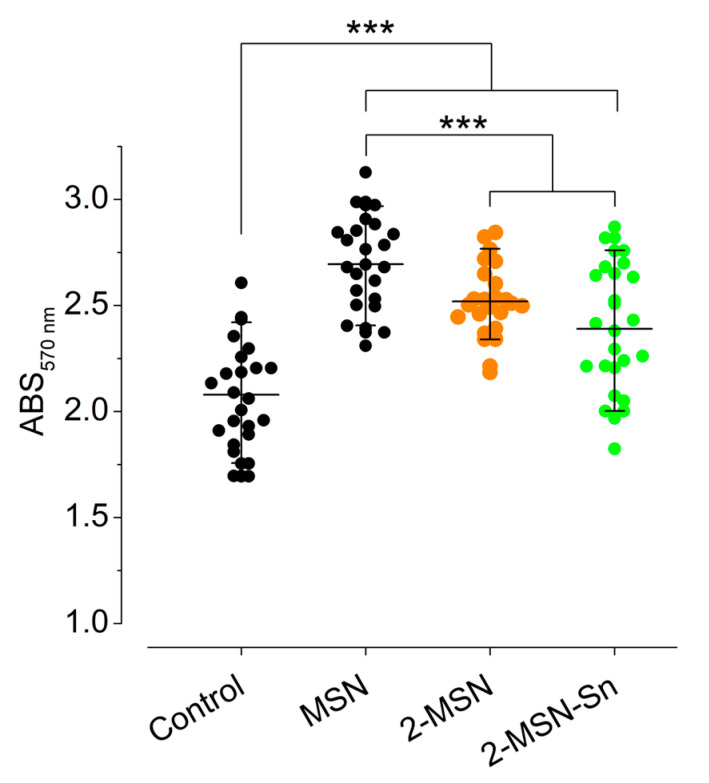
*S. aureus* biofilm-nanoparticle interaction of **MSN**, **2-MSN** and **2-MSN-Sn** materials at 250 µg/mL. *** *p*-value < 0.001 from Mann–Whitney test. The bars indicate median and interquartile range.

Nanoparticle interaction with a mature *S. aureus* biofilm was analyzed upon incubation for 30 min with 250 µg/mL of **MSN**, **2-MSN** or **2-MSN-Sn**. As observed in Figure 5, the highest retention values were observed for the non-functionalized **MSN**, while the two functionalized materials showed lower values and no significant differences between them. Additionally, the absorbance value of the nanoparticle-treated cultures was above the value obtained for the control experiment, indicating that **MSN**, **2-MSN** and **2-MSN-Sn** can interact with the biofilm and keep adhered to it.

#### 3.2.6. Inhibition of Adherence Stage during the Biofilm Formation and during the Biofilm Development

**Figure 6:**
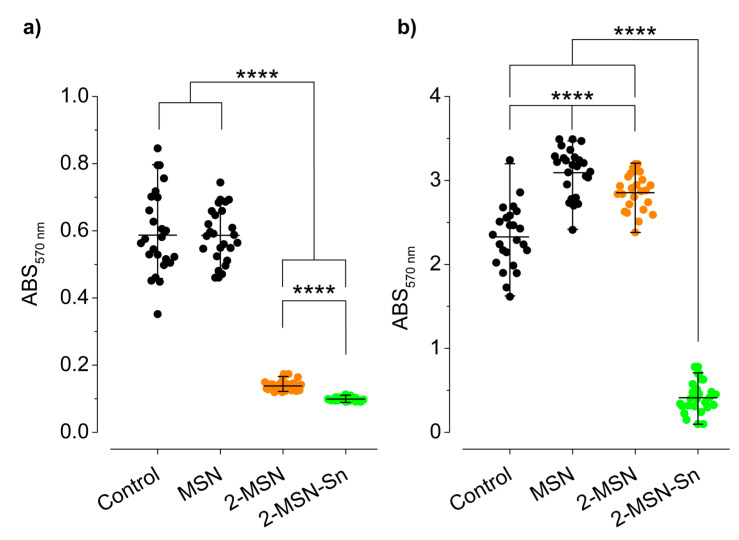
Inhibition of (**a**) attachment and (**b**) biofilm development of **MSN**, **2-MSN** and **2-MSN-Sn** materials at 250 µg/mL. **** *p*-value < 0.0001 from Mann–Whitney tests. The bars indicate median and interquartile range.

The ability of the nanoparticles to inhibit the stages of attachment, Figure 6a, and biofilm development, Figure 6b, was analyzed.

As observed in Figure 6a, the unmodified material (**MSN**) was unable to inhibit bacterial attachment, possibly due to the absence of the bioactive fragments (organotin(IV) compound and coumarin343) on the surface of the system. However, **2-MSN** and **2-MSN-Sn** effectively precluded the attachment of bacteria to the surface, inhibiting the process in a 75.9 and 82.8%, respectively. The presence of the organotin(IV) cytotoxic agent in **2-MSN-Sn** (as opposed to **2-MSN**) significantly decreased ca. 6.9% the adherence of *S. aureus*.

With respect to the inhibition of the biofilm development stage, Figure 6b, activity was only observed for the fully functionalized material **2-MSN-Sn which** was able to inhibit 85.6% the *S. aureus* biofilm development. Therefore, it can be concluded that the observed inhibition on *S. aureus* biofilm development is a consequence of the incorporation of the organotin(IV) compound in the system.

### 3.3. Evaluation of In Vitro Imaging Capability in the Biological Environment

After the evaluation of the anticancer and antibacterial activity of the final materials, the ability of the systems to perform their diagnostic function by the emission of light resulting from the functionalization with the imaging agent (natural derivative of coumarin) was evaluated. For this purpose, the ability of the materials to internalize in cancer cells and the efficacy of the **2-MSN-Sn** material to inhibit the biofilm development stage were studied.

#### 3.3.1. Cell Uptake Studies by Confocal Microscopy

**Figure 7:**
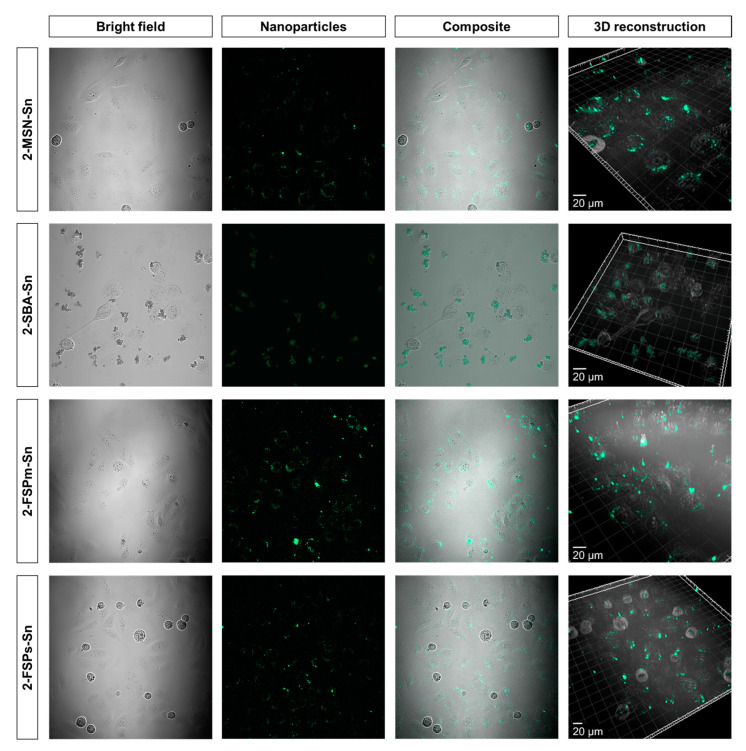
CLSM images of MDA-MB-231 cells incubated with **2-MSN-Sn**, **2-SBA-Sn**, **2-FSPm-Sn** and **2-FSPs-Sn**. Excitation wavelength 443 nm and emission wavelength 480 nm.

To determine compound internalization, confocal laser scanning microscopy (CLSM) images were obtained from a culture of MDA-MB-231 cells incubated with **2-MSN-Sn**, **2-SBA-Sn**, **2-FSPm-Sn** and **2-FSPs-Sn**. The images, shown in Figure 7, were obtained by applying the determined excitation and emission parameters (see Figures S8 and S9 for more details).

As observed in Figure 7, green light emission is detected inside tumor cells upon incubation with **2-MSN-Sn**, **2-FSPm-Sn** and **2-FSPs-Sn**, indicative of a correct internalization to the cytoplasm, as concluded from the green circular organelles observed. The spherical structures observed in the micrographs correspond to non-viable MDA-MB-231 cells, which confirms the cytotoxic capacity of the materials.

On the contrary, in the pictures taken for the study with **2-SBA-Sn** material, it was observed that several clusters of particles accumulated outside of the cells, confirming that the SBA-15-based system was not uptaken by tumor cells and remained accumulated in the extracellular medium. It is important to note that **2-SBA-Sn** is the material with the largest particle size and may not easily be able to internalize in cells.

#### 3.3.2. Visualization of Inhibition of Biofilm Development by Optical and Fluorescence Microscopy

**Figure 8:**
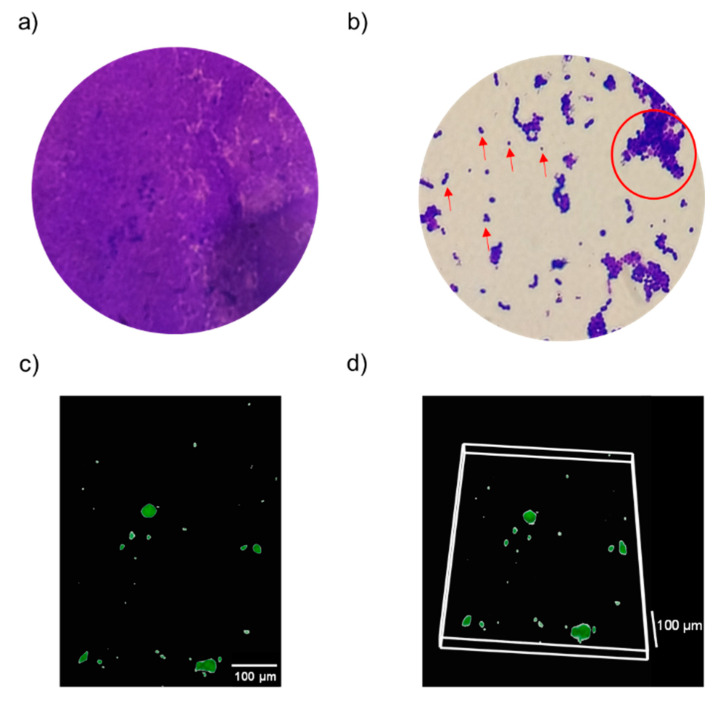
(**a**) Optical microscopy image of the *S. aureus* biofilm in the absence of NPs (100×), and (**b**) optical microscopy (100×), (**c**) fluorescence microscopy images XY plane, and (**d**) 3D reconstruction after inhibition of biofilm development with **2-MSN-Sn** (250 µg/mL). Excitation wavelength 490 nm and emission wavelength 519 nm. Red arrows indicate individual bacteria and red circle indicate biofilm fragments.

The high efficacy of **2-MSN-Sn** in inhibiting the development of biofilm was confirmed by optical and fluorescence microscopy (Figure 8).

A comparison of the optical microscopy images obtained (Figure 8a,b) show that the biofilm changes from exhibiting a large amount of substance and a high apparent density to being clearly disaggregated, with only small biofilm fragments (red circle) and individual bacteria (red arrows), after treatment with **2-MSN-Sn**. Furthermore, the presence of coumarin343 in **2-MSN-Sn** allowed the study of the biofilm development inhibition by fluorescence microscopy (Figure 8c,d). In this regard, the 2D and 3D images showed a few fluorescent areas, thus demonstrating that, after treatment, only a minimal portion of the biofilm remained intact.

## 4. Conclusions

The work presented herein has focused on the design of four systems based on different starting silicas (MSN, SBA-15, FSPs and FSPm) for their analysis as non-classical drug delivery systems against triple negative breast cancer MDA-MB-231 and *S. aureus* infection. Silicas functionalized with a derivative of natural coumarin as an imaging agent and natural folic acid as a target molecule, show that only 5% in weight of each agent was enough for high selectivity and marked improvement in cytotoxic activity. The comparison of the final materials showed that the morphological and size differences of the silica influence the successive functionalization steps, observing variations in the structural parameters and incorporated quantities. In addition, differences in biological activities have also been observed. In the field of anticancer agents, all materials show very significant and promising activity. However, only the silica-based materials with ordered internal structures (**2-MSN-Sn** and **2-SBA-Sn**) showed selectivity towards cancer cells. In this context, in the area of antibacterial agents, material **2-MSN-Sn** was the only system that showed bacteriostatic activity against *S. aureus*, was able to attach on a *S. aureus* biofilm and inhibit the *S. aureus* biofilm development. This points towards a high potential of this system in the treatment of chronic wounds, which will be one of the main objectives for future applications of this nanomaterial. The antibacterial properties against planktonic bacteria of **2-MSN-Sn** come from the promotion of high oxidative stress, as **2-MSN-Sn** significantly increased ROS levels in the cell even at low concentrations. In summary, we have prepared, characterized, and identified a dual application theranostic agent for TNBC and *S. aureus* bacterial infections (biofilm) which can now be studied in preclinical trials in animal models.

## Figures and Tables

**Table 1:** Textural parameters obtained by nitrogen adsorption/desorption isotherms.

Material	BET Surface (m^2^/g)	Pore Volume (cm^3^/g)	Pore Diameter (nm)
**MSN**	1042	1.04	3.81
**1-MSN-Sn**	691	0.41	2.06
**2-MSN-Sn**	702	0.78	2.45
**SBA-15**	650	0.80	4.74
**2-SBA-Sn**	394	0.51	4.08
**FSPm**	317	0.49	5.80
**2-FSPm-Sn**	132	0.23	-
**FSPs**	315	0.69	8.91
**2-FSPs-Sn**	168	0.45	-

**Table 2:** Determination of the quantity of ligand and tin by thermogravimetry or ICP-MS, respectively.

Material	%AP	%COU + FA	Experimental Sn wt%
**1-MSN-Sn**	5.0	10.1	7.2
**2-MSN-Sn**	5.0	2.4	2.0
**2-SBA-Sn**	8.5	3.2	0.9
**2-FSPm-Sn**	2.0	3.5	0.5
**2-FSPs-Sn**	5.0	3.7	0.7

**Table 3:** XRD data of **MSN**, **SBA-15** and all their functionalized materials.

Material	*hkl*	2θ (°)	d*_hkl_* (nm)	*a*_0_ (nm)
**MSN**	100	2.45	3.60	4.16
	110	4.12	2.14	2.48
	200	4.67	1.89	2.18
**MSN-AP**	100	2.45	3.60	4.16
**1-MSN**	100	2.56	3.46	4.00
**1-MSN-Sn**	100	2.61	3.39	3.91
**2-MSN**	100	2.53	3.49	4.03
**2-MSN-Sn**	100	2.53	3.49	4.03
**SBA-15**	100	1.18	7.50	8.66
	110	1.91	4.63	5.35
	200	2.17	4.08	4.70
**SBA-AP**	100	1.21	7.34	8.47
**2-SBA**	100	1.18	7.50	8.66
**2-SBA-Sn**	100	1.20	7.34	8.47

**Table 4:** Cytotoxic activity values of all the final materials calculated as a IC_50_ function of the metallic load (µM).

Material	IC_50_ µM vs. [Sn]	
	HEK-293T	MDA-MB-231
**1-MSN-Sn**	2.11 ± 0.05	7.73 ± 0.24
**2-MSN-Sn**	1.63 ± 0.52	0.69 ± 0.31
**2-SBA-Sn**	5.31 ± 1.94	0.81 ± 0.05
**2-FSPm-Sn**	0.18 ± 1.06	0.44 ± 0.03
**2-FSPs-Sn**	0.91 ± 0.50	0.83 ± 0.42

**Table 5:** Minimum inhibitory concentration (MIC) and bactericidal concentration (MBC) for final materials against planktonic *S. aureus* strain. Data are given in µg/mL of material and data in brackets refer to µg/mL of tin.

Material	ATCC29213	
	MIC	MBC
**1-MSN-Sn**	>2000 (>144.0)	>2000 (>144.0)
**2-MSN-Sn**	125 (2.5)	2000 (40.0)
**2-SBA-Sn**	>2000 (>17.0)	>2000 (>17.0)
**2-FSPm-Sn**	>2000 (>10.0)	>2000 (>10.0)
**2-FSPs-Sn**	>2000 (>14.0)	>2000 (>14.0)

## Data Availability

Data are available on request.

## Supplementary Materials

The following supporting information can be downloaded at: https://www.mdpi.com/article/10.3390/pharmaceutics15020560/s1, Figure S1: Particle size distributions of the materials (a) **1-MSN-Sn**, (b) **2-MSN-Sn**, (c) **2-SBA-Sn**, (d) **2-FSPm-Sn** and (e) **2-FSPs-Sn**; Figure S2: Nitrogen adsorption (solid line, →) and desorption (dashed line, ←) isotherms of materials based on (a) **MSN**, (b) **SBA-15**, (c) **FSPm** and (d) **FSPs**; black line: non-functionalized nanoparticles and green line: functionalized final materials; Figure S3: Pore size distribution of organotin-functionalized materials (a) **1-MSN-Sn** and **2-MSN-Sn**, (b) **2-SBA-Sn**, (c) **2-FSPm-Sn** and (d) **2-FSPs-Sn**; Figure S4: TG of materials based on based on (a) **MSN**, (b) **SBA-15**, (c) **FSPm** and (d) **FSPs**; Figure S5: FT-IR spectra of the final materials and their respective starting silica; Figure S6: DR-UV spectra of (a) **1-MSN-Sn** and **2-MSN-Sn**, (b) **2-SBA-Sn**, (c) **2-FSPm-Sn** and (d) **2-FSPs-Sn**; Figure S7: ^13^C CP MAS NMR spectra of (a) **1-MSN-Sn** and **2-MSN-Sn**, (b) **2-SBA-Sn**, (c) **2-FSPm-Sn** and (d) **2-FSPs-Sn**; Figure S8: Experimental solid-state excitation (solid line) and emission (dashed line) spectra of coumarin343 (black) and **1-MSN-Sn** (green). Figure S9: Solid-state photoluminescence excitation and emission spectra of (a) **2-MSN-Sn**, (b) **2-SBA-Sn**, (c) **2-FPSm-Sn** and (d) **2-FSPs-Sn**; Figure S10: Cell viability of starting (black) and final (green) materials of the systems based on (a) **MSN**, (b) **SBA-15**, (c) **FSPm** and (d) **FSPs** against MDA-MB-231 cell line; values = mean ± SD, *n* = 3 independent experiments and 3 replicates/experiment; Figure S11: Cell viability of starting (black) and final (green) materials of the systems based on (a) **MSN**, (b) **SBA-15**, (c) **FSPm** and (d) **FSPs** against HEK-293T cell line; values = mean ± SD, *n* = 3 independent experiments and 3 replicates/experiment; Table S1: Values of the cytotoxic activity of the starting silicas, calculated as a function of the IC_50_ of all the material (µg/mL); Table S2: Comparison of minimum inhibitory concentration (MIC) and bactericidal concentration (MBC) for final materials against planktonic ATCC29213 *S. aureus* strain. References [83,84,85] are cited in the Supplementary Materials.
