# Supplementary material for: Dual Anticancer and Antibacterial Properties of Silica-Based Theranostic Nanomaterials Functionalized with Coumarin343, Folic Acid and a Cytotoxic Organotin(IV) Metallodrug
Source: Pharmaceutics. 2023 Feb 7;15(2):560. doi: 10.3390/pharmaceutics15020560 (PMC9962538; doi:10.3390/pharmaceutics15020560)
Supplement: Supplementary file 1 [file pharmaceutics-15-00560-s001.zip › pharmaceutics-2149032-supplementary.pdf]

SUPPORTING INFORMATION

Dual Anticancer and Antibacterial Properties of Silica-Based Theranostic Nanomaterials Functionalized With Coumarin343, Folic Acid and a Cytotoxic Organotin(IV) Metallodrug

1. Physicochemical characterization of functionalized NPs.
    a. Particle size distribution of the final materials
    b. Textural properties. Nitrogen adsorption-desorption isotherms and pore size distribution of the starting and the final materials
    c. TG of the functionalized materials.
    d. FTIR and UV-Vis spectroscopy
    e. Solid State RMN
    f. Analysis of the image agent by photoluminescence studies
2. *In Vitro* Studies of anticancer activity
    a. Cell viability studies
3. Antibacterial Studies
    a. MIC and MBC Comparison Table

1. Physicochemical characterization of functionalized NPs.

a. Particle size distribution of the final materials

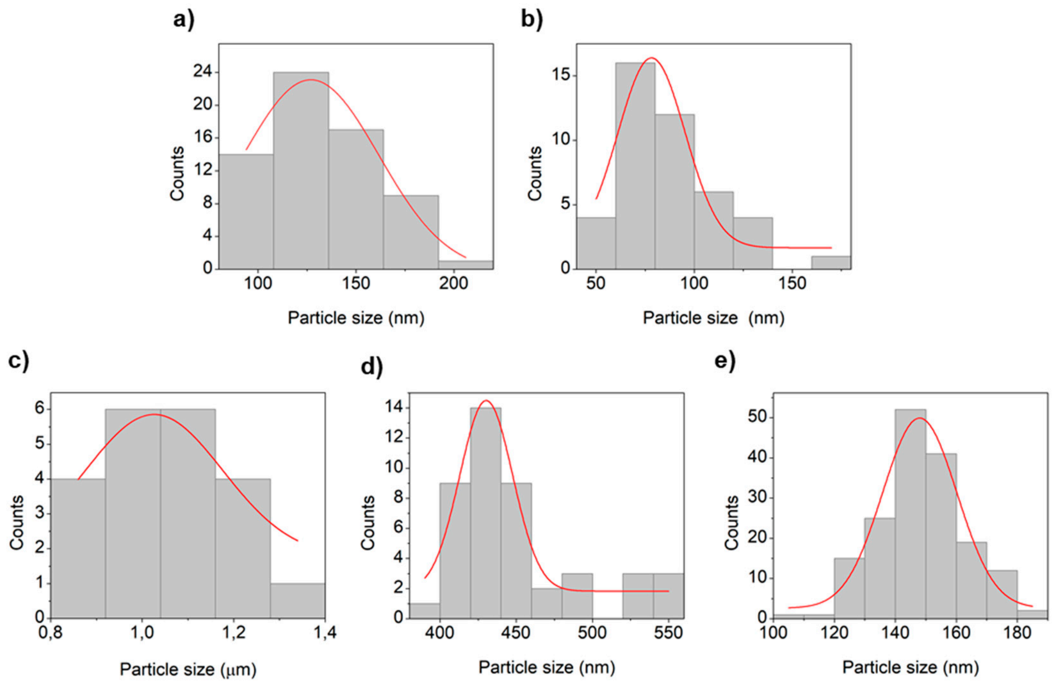

Figure S1. Particle size distributions of the materials a) **1-MSN-Sn**, b) **2-MSN-Sn**, c) **2-SBA-Sn**, d) **2-FSPm-Sn** and e) **2-FSPs-Sn.**

b. Nitrogen adsorption-desorption isotherms and pore size distribution

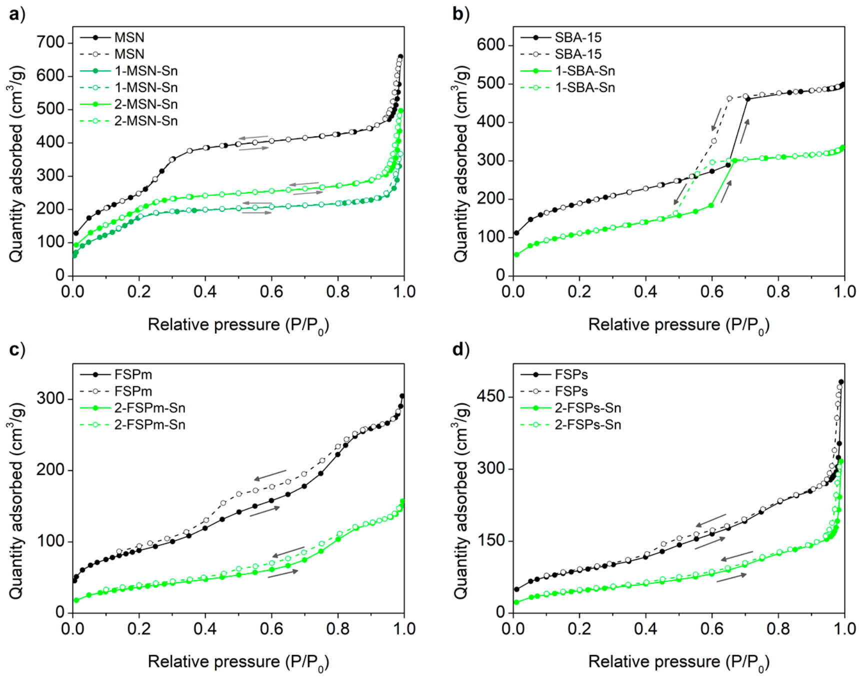

Figure S2. Nitrogen adsorption (solid line, →) and desorption (dashed line, ←) isotherms of materials based on a) **MSN**, b) **SBA-15**, c) **FSPm** and d) **FSPs**; black line: non-functionalized nanoparticles and green line: functionalized final materials.

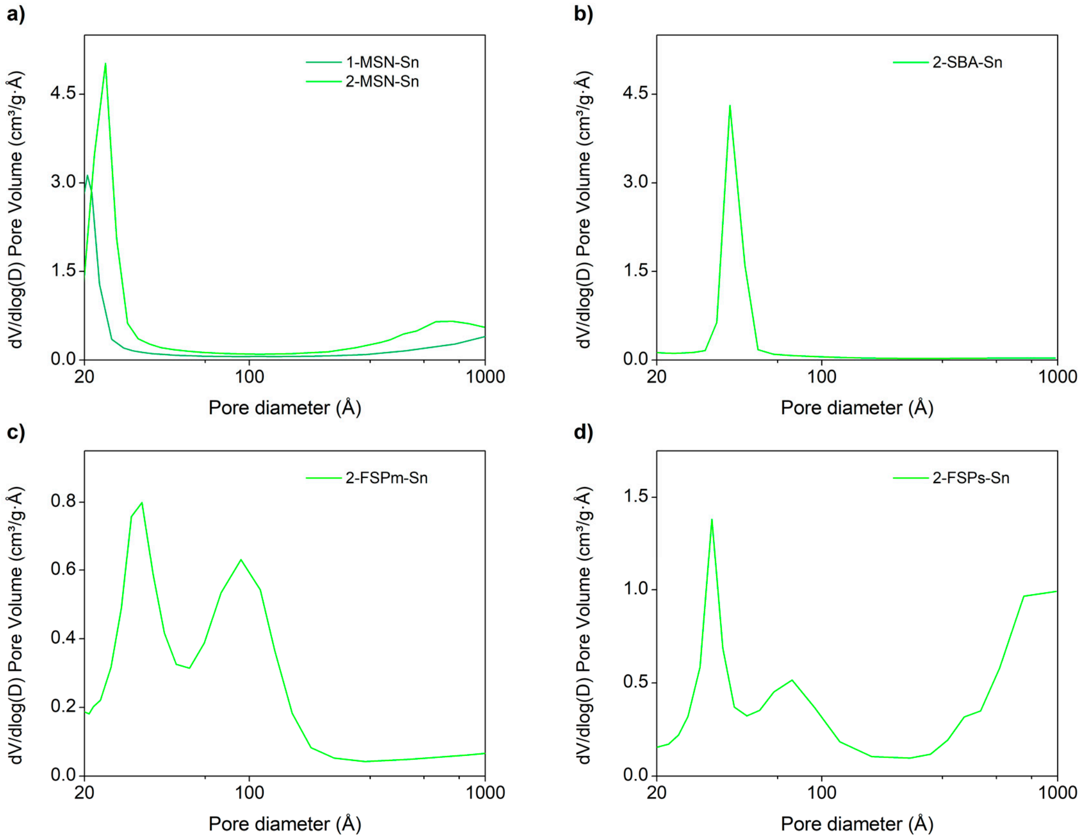

Figure S3. Pore size distribution of organotin-functionalized materials a) **1-MSN-Sn** and **2-MSN-Sn**, b) **2-SBA-Sn**, c) **2-FSPm-Sn** and d) **2-FSPs-Sn**.

c. TG of the functionalized materials

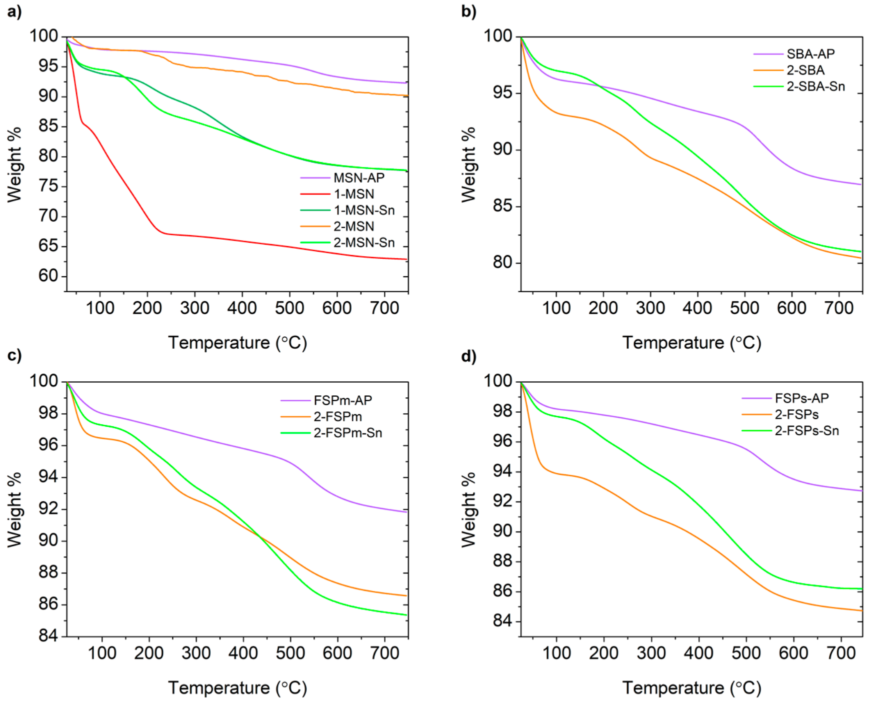

Figure S4. TG of materials based on based on a) **MSN**, b) **SBA-15**, c) **FSPm** and d) **FSPs**.

d. FT-IR and UV-Vis spectroscopy

The final materials were characterized by infrared (FT-IR) and diffuse reflectance ultraviolet spectroscopy (DR-UV). FT-IR spectra showed the typical signals of silica functionalized with coumarin, folic acid and the organotin complex (Figure S5). The spectra presented broad bands between 3500 and 3200 cm^{-1} (O–H stretching of the silanol groups and the adsorbed water molecules), a broad band around 1100 cm^{-1} (assigned to the siloxane (Si–O–Si) groups), the stretching band of the Si–O bonds at ca. 900 cm^{-1} and the deformation vibrations of adsorbed water molecules at ca. 1630 cm^{-1}. In addition of the signals of the silica, a set of less intense bands were observed in 3100 and 2800 cm^{-1} region which can be associated with C–H and N–H vibrations of the different ligands. Finally, a bunch of lower intense bands located in the 1700 and 1300 cm^{-1} and 690-735 cm^{-1} regions can be ascribed to the amido and carbonyl groups.

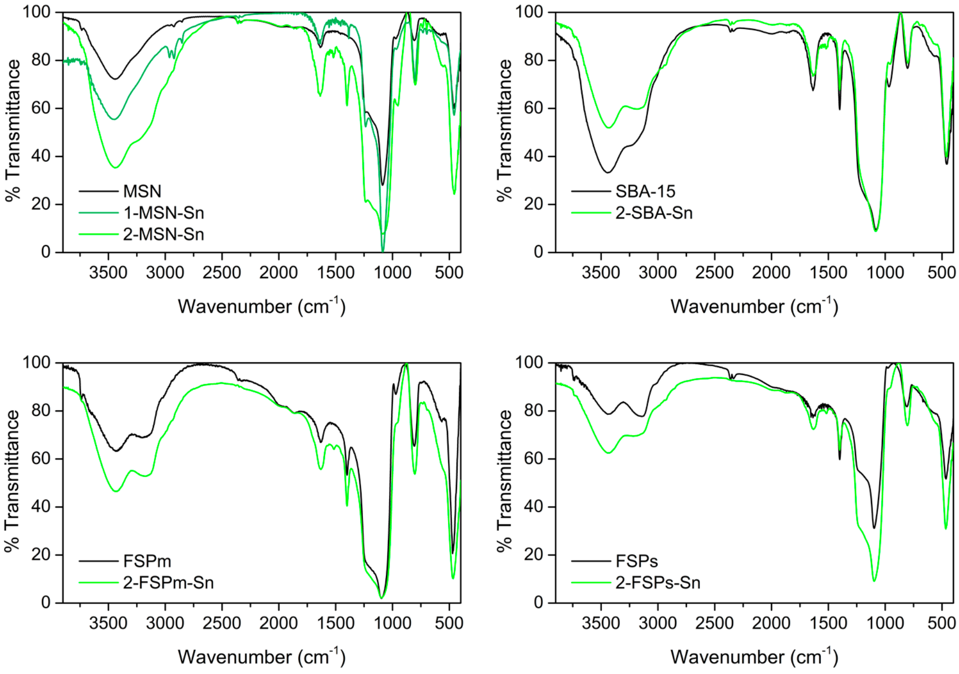

Figure S5. FT-IR spectra of the final materials and their respective starting silica.

The ultraviolet spectroscopy (DR-UV) of **1-MSN-Sn** (Figure S6) showed an intense absorption peak around 220 nm associated to the amino ligand AP and a smaller peak at ca. 260 nm corresponding to the incorporation of the organotin derivative.[1] Additionally, the spectra showed a low intensity shoulder at ca. 290 nm, which can be associated with the folic acid and coumarin of the material. Furthermore, the spectra contain two additional bands around 365 nm and 460 nm assigned respectively to folic acid and coumarin343 fragments. Similar spectra were also observed for the other silica nanocarriers (Figure S6). In this case, due to the low tin percentage, the 260 nm organotin bands may be note visible by their overlap with the 290 nm band corresponding to the FA and COU fragments.

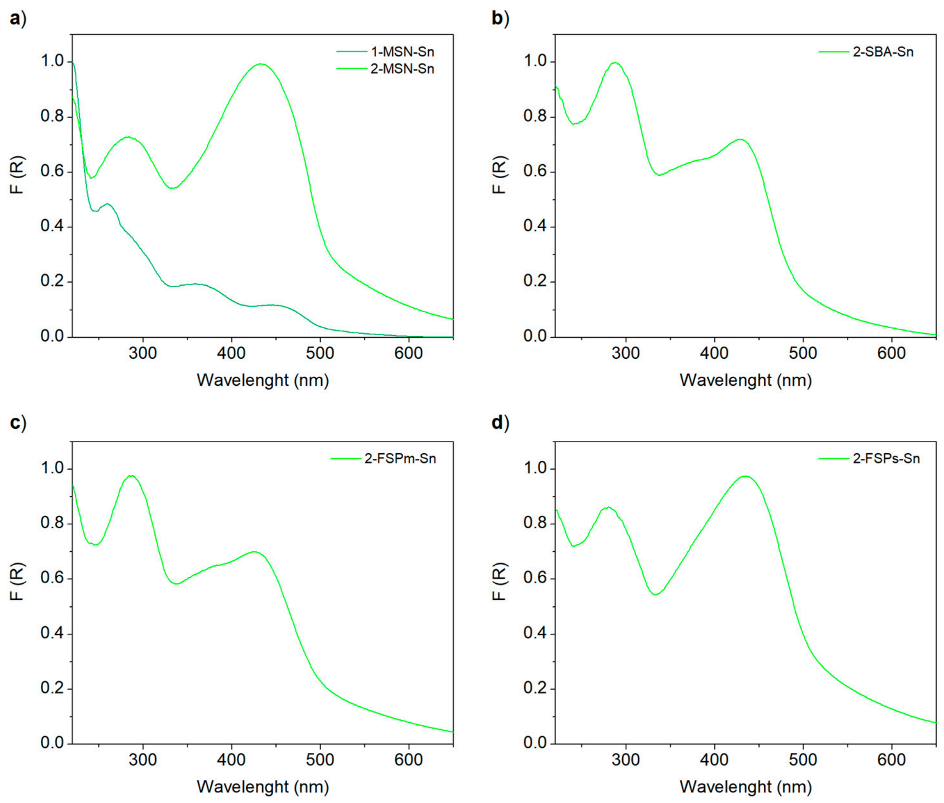

Figure S6. DR-UV spectra of a) **1-MSN-Sn** and **2-MSN-Sn**, b) **2-SBA-Sn**, c) **2-FPSm-Sn** and d) **2-FSPs-Sn**.

e. Solid-state NMR spectroscopy

In order to test the adequate functionalization of the materials with AP, coumarin343, folic acid and the organotin(IV) compound, tin-functionalized materials have been characterized by ^{13}C CP MAS spectroscopy. (Figure S7). The ^{13}C CP MAS spectra of all systems showed a set of signals between 0 and 60 ppm. These peaks are due to the functionalization of silica with AP because of the aliphatic carbons of the chain. In addition, the peaks representative of the $SCH_2CH_2CH_2CH_2Si$ chain of the organotin(IV)-containing fragment also appeared in this range.

Additionally, a set of signals were observed in 100 - 150 ppm region and were associated with the carbon atoms of the aromatic rings of coumarin343 and folic acid and the $SnPh_3$ fragment. Finally, another set of signals were observed between 150 and 200 ppm and were attributed to the C=O carbons of folic acid and coumarin, and to the aromatic C–N of folic acid.

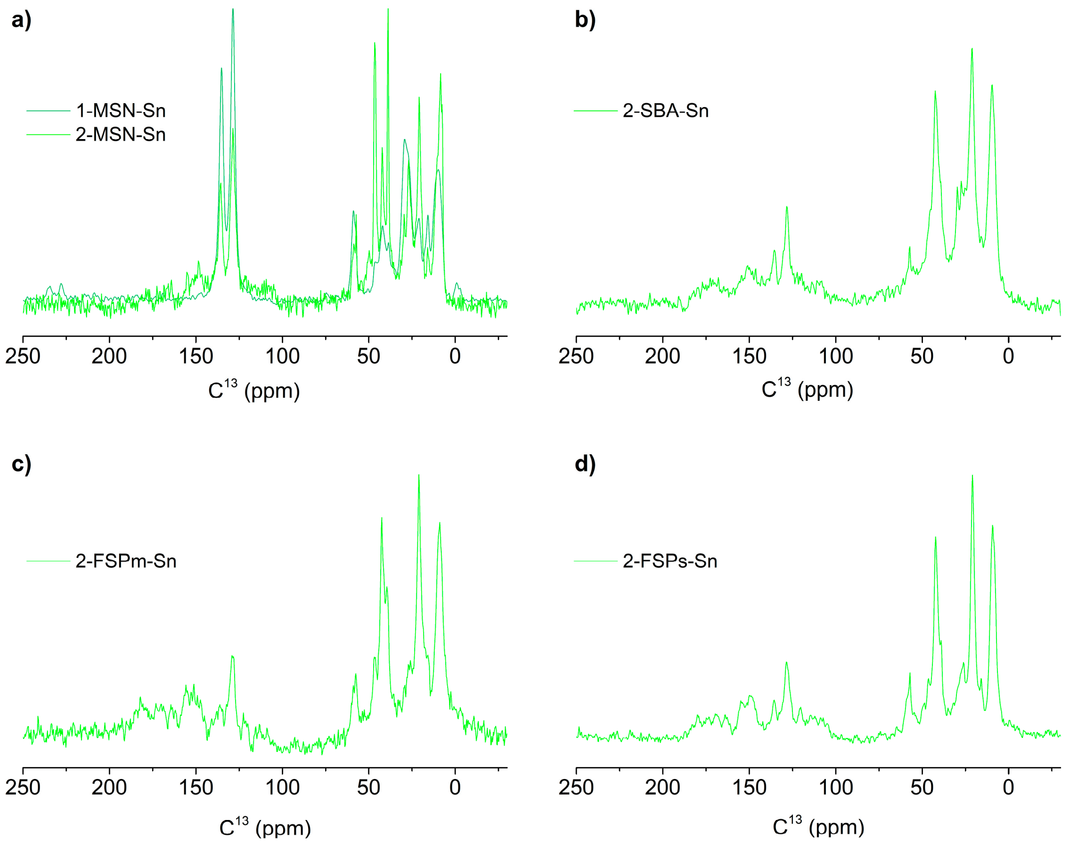

Figure S7. ^{13}C CP MAS NMR spectra of a) **1-MSN-Sn** and **2-MSN-Sn**, b) **2-SBA-Sn**, c) **2-FPSm-Sn** and d) **2-FSPs-Sn**.

f. Analysis of the imaging agent by photoluminescence studies

Photoluminescence studies performed in functionalized materials further allowed the determination of the successful functionalization with coumarin343. The studied materials showed photoluminescent properties. Excitation and emission spectra were recorded in solid-state samples at room temperature. Coumarin343 is a fluorophore and in the solid state has a maximum absorption peak at ca. 500 nm and a maximum emission peak at 570 nm (Figure S8). As can be observed in Figure S8 the emission spectrum of **1-MSN-Sn** shows a broad band with the maximum at 493 nm. This band could be assigned to the $\pi \rightarrow \pi^*$ transition corresponding to the organic ligands. The excitation spectrum also shows a broad band at 350-475 nm with a maximum at 446 nm. The materials **2-MSN Sn**, **2-SBA-Sn**, **2-FSPm-Sn** and **2-FSPs-Sn** as can be seen in Figure S8, showed similar results. In any case, in all the materials it was observed that the excitation and emission maxima underwent a significant hypochromic shift of about 100 nm, i.e. they were shifted to shorter wavelengths compared with free coumarin343. This phenomenon may be occurring due to the functionalization of coumarin with mesoporous silica. Several examples supporting this evidence have been found in the literature.[68,69]

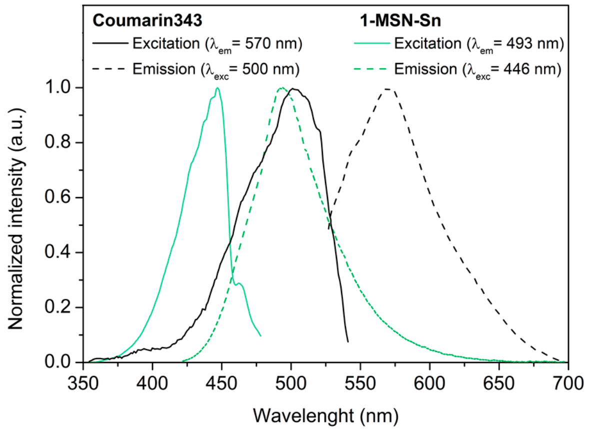

Figure S8. Experimental solid-state excitation (solid line) and emission (dashed line) spectra of coumarin343 (black) and **1-MSN-Sn** (green).

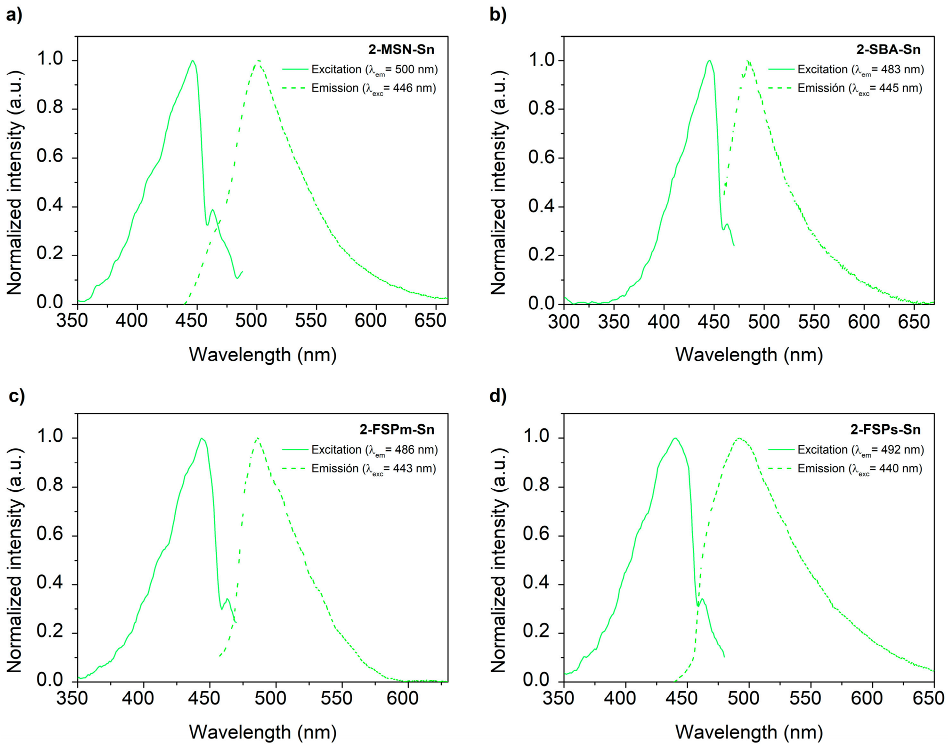

Figure S9. Solid-state photoluminescence excitation and emission spectra of a) **2-MSN-Sn**, b) **2-SBA-Sn**, c) **2-FPSm-Sn** and d) **2-FSPs-Sn**.

2. *In Vitro Studies of anticancer activity*

a. Cell viability studies

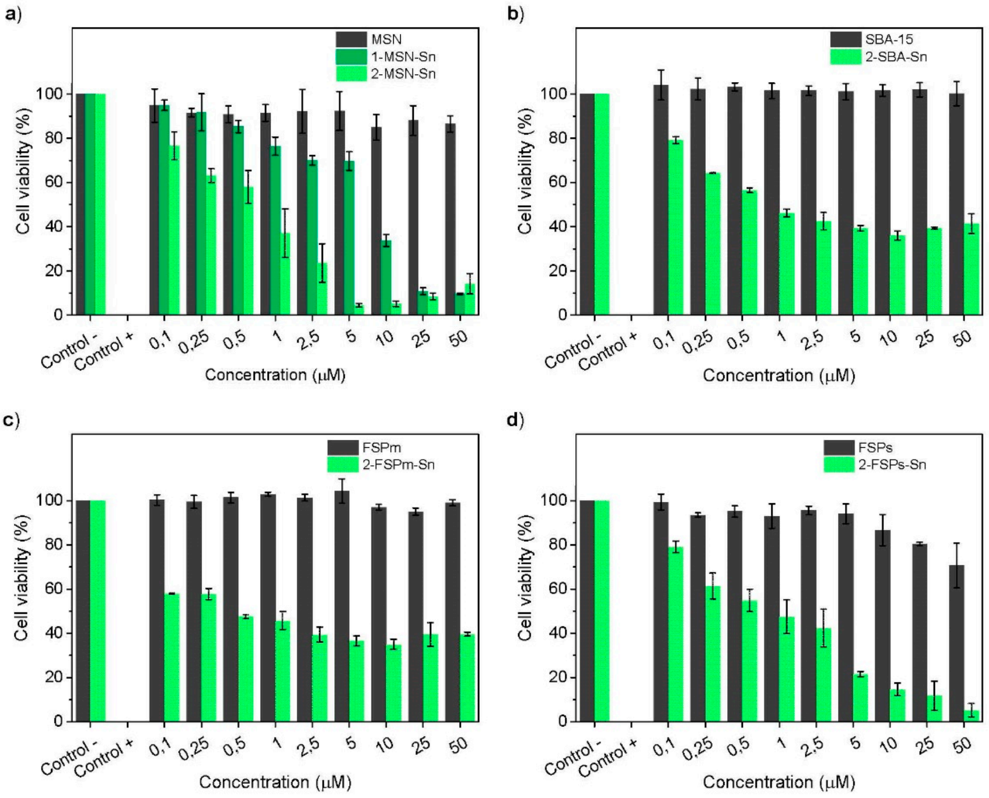

Figure S10. Cell viability of starting (black) and final (green) materials of the systems based on a) **MSN**, b) **SBA-15**, c) **FSPm** and d) **FSPs** against MDA-MB-231 cell line; values = mean ±SD, n=3 independent experiments and 3 replicates/experiment.

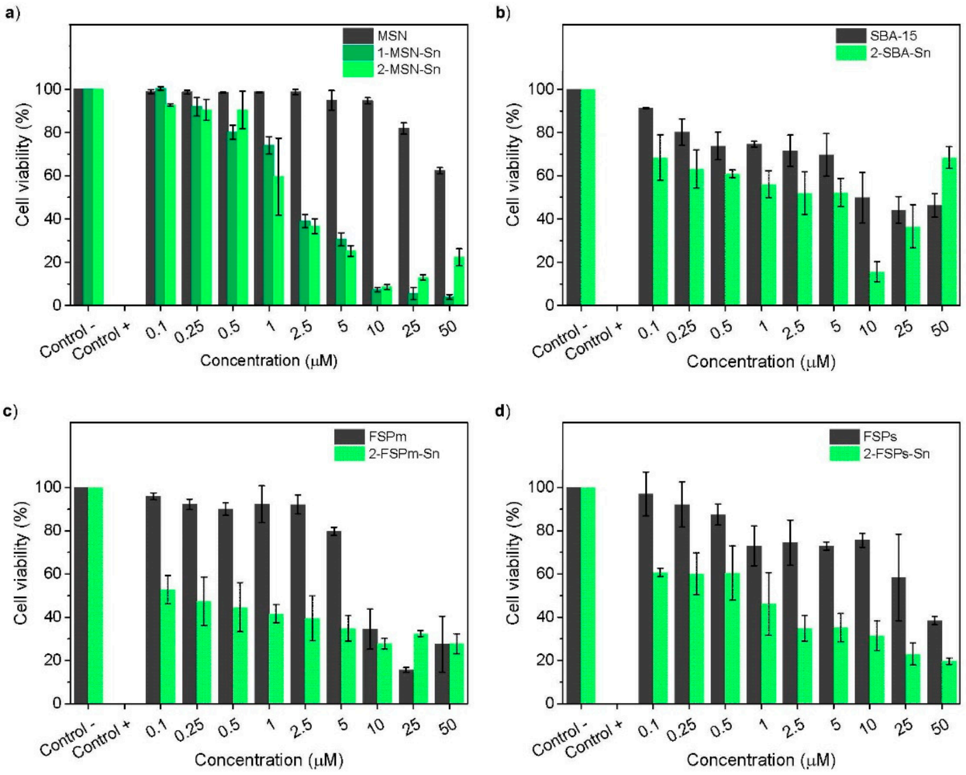

Figure S11. Cell viability of starting (black) and final (green) materials of the systems based on a) **MSN**, b) **SBA-15**, c) **FSPm** and d) **FSPs** against HEK-293T cell line; values = mean ±SD, n=3 independent experiments and 3 replicates/experiment.

Table S1. Values of the cytotoxic activity of the starting silicas, calculated as a function of the IC_{50} of all the material (μg/mL).

**Material**	**IC_{50} μg/mL vs [material]**	
	**HEK-293T**	**MDA-MB-231**
**MSN**	-	-
**SBA-15**	131.50 ± 16.26	-
**FSPm**	196.82 ± 18.99	-
**FSPs**	602.19 ± 44.94	-

3. Antibacterial Studies

   a. MIC and MBC Comparison Table

Table S2. Comparison of minimum inhibitory concentration (MIC) and bactericidal concentration (MBC) for final materials against planktonic ATCC29213 *S. aureus* strain. Data are given in μg/mL of material and data in brackets refer to μg/mL of tin.

Material	ATCC29213	
	**MIC**	**MBC**
**1-MSN-Sn**	> 2000 (>144.0)	> 2000 (>144.0)
**2-MSN-Sn**	125 (2.5)	2000 (40.0)
**2-SBA-Sn**	> 2000 (>17.0)	> 2000 (>17.0)
**2-FSPm-Sn**	> 2000 (>10.0)	> 2000 (>10.0)
**2-FSPs-Sn**	> 2000 (>14.0)	> 2000 (>14.0)
**ciprofloxacin**	0.5	0.5
**gentamicin**	1.0	1.0
**rifampicin**	0.2	>0.8

References for ciprofloxacin, gentamicin and rifampicin: Quinteros et al. Toxicology in Vitro 36 (2016) 216–223; Bustos et al. Food and Chemical Toxicology 118 (2018) 294–302; Scolari et al. Drug Delivery and Translational Research 10 (2020) 1403–1417

References

1. Quinteros, M.A.; Aristizábal, V.C.; Dalmasso, P.R.; Paraje, M.G.; Páez, P.L. Oxidative stress generation of silver nanoparticles in three bacterial genera and its relationship with the antimicrobial activity. *Toxicol. Vitr.* **2016**, *36*, 216–223.

2. Bustos, P.S.; Deza-Ponzio, R.; Páez, P.L.; Cabrera, J.L.; Virgolini, M.B.; Ortega, M.G. Flavonoids as protective agents against oxidative stress induced by gentamicin in systemic circulation. Potent protective activity and microbial synergism of luteolin. *Food Chem. Toxicol.* **2018**, *118*, 294–302.

3. Scolari, I.R.; Páez, P.L.; Musri, M.M.; Petiti, J.P.; Torres, A.; Granero, G.E. Rifampicin loaded in alginate/chitosan nanoparticles as a promising pulmonary carrier against Staphylococcus aureus. *Drug Deliv. Transl. Res.* **2020**, *10*, 1403–1417.
